# Over and over Again

**Published:** 1892-10

**Authors:** 


### OVER AND OVER AGAIN.

Over and over again,
  No matter which way I may turn,
I always find in the book of life
  Some lessons I have to learn.
One doing will not suffice,
  Though doing is not in vain ;
And a blessing failing me once or twice,
  May come if I try again.

The path that has once been trod
  Is never so rough to the feet ;
And the lesson we once have learned
  Is never so hard to repeat.
Though sorrowful tears may fall,
  And the heart to its depths be riven
By storm and tempest we heed them all
  To render us fit for heaven.—*Ex.*

